# Investigating the slice thickness effect on noise and diagnostic content of single-source multi-slice computerized axial tomography

**DOI:** 10.25122/jml-2022-0188

**Published:** 2023-06

**Authors:** Nashwan Karkhi Abdulkareem, Shereen Ismail Hajee, Fatiheea Fatihalla Hassan, Ilham Khalid Ibrahim, Ruaa Emad Hussein Al-Khalidi, Noor Abubaker Abdulqader

**Affiliations:** 1 Department of Pharmacology and Medical Physics and Clinical Biochemistry, College of Medicine, Hawler Medical University, Erbil, Iraq; 2 Department of Radiology, Erbil Technical Medical Institute, Erbil Polytechnic University, Erbil, Iraq; 3 Department of Radiology, Rozh Halat Emergency Hospital, Ministry of Health, Erbil, Iraq

**Keywords:** image noise, single-source computed tomography, slice thickness, image quality

## Abstract

High-quality and detailed CT scan images are crucial for accurate diagnosis. Factors such as image noise and slice thickness affect image quality. This study aimed to determine the optimal slice thickness that minimized image noise while maintaining sufficient diagnostic information using the single-source computed tomography head protocol. Single-source CT images were examined using the Linux Operating system Ge Revolution 64-slice CT scanner, and a combination of statical analysis and DICOM CT image analysis was employed. The single-source energy head CT protocol was used to investigate the effect of slice thickness on noise and visibility in images. Different values of slice thickness 0.625, 1.25, 2.5, 3.75, 5, 7.5, and 10 were prepared, and then quantitative analysis was performed. Thinner slice thickness decreased image noise, increased visibility, and improved detection. Therefore, the balance between changing the thickness of the slice with the diagnostic content and image noise must be considered. Maximum slice thickness enhances CT image detail and structure despite more noise. Based on the results, a slice thickness of 1.25mm was identified as the optimal choice for reducing image noise and achieving better and more accurate detection using the single-source computed tomography head protocol. The study revealed that image noise tends to increase with greater slice thickness according to the Linux operating system. These findings can serve as a valuable guide for quality control methods in CT centers, emphasizing the need to determine the appropriate slice thickness to ensure an accurate diagnosis.

## INTRODUCTION

The introduction of computed tomography (CT) scans in the late 1970s revolutionized medical imaging and diagnostic methods [1]. CT scans utilize X-rays to generate cross-sectional images or slices of the target organ [2]. These sectional images are reconstructed by measuring the attenuation coefficient of X-rays in the volume of the object under study [3]. Advancements in CT scan devices, including spiral imaging features, multi-slice capability, increased energy supply, and improved detectors, have continuously enhanced image quality and diagnostic value [1]. Currently, 64-slice devices with a single source are widely used, resulting in improved resolution, instant resolution, speed, and accuracy of images [4, 5]. These devices use a single polychromatic X-ray beam (80 to 140 Kv with a standard of 120 Kv) emitted from a single source and received by two-row detectors and 64 slices of the X-ray source. Since the images depend on the absorption of X-rays (related to the voltage applied to the tube), the resulting images depend on energy (or voltage). In these devices, the maximum and minimum tube voltages are 140 and 80 kVp, respectively, resulting in an energy difference of up to 60 kVp [6]. However, since the ray group is a spectrum of energies with the specific energy of the anode constituent, the average energy of the two energy spectra can be 76 and 56 keV, in which the energy difference is smaller [7].

The quality of images in CT scans is determined by three parameters: image contrast, spatial resolution, and noise [2]. Noise, defined as the standard deviation of CT numbers in a uniform image, represents unwanted information in the intended signal of electronic systems and comes from many sources, including electronic interference. It also appears as an irregular grain pattern in all images, reducing image quality and information [8]. The noise level can be reduced by lowering the standard deviation of pixel values. It is further defined by detectors (quantum noise) depending on the number of photons received. The image noise decreases as the number of photons received increases [9]. In CT systems, the amount of noise is inversely related to resolution, with spatial resolution referring to its ability to display images of closely positioned objects as separate entities [10]. It is possible to reduce the image noise without increasing the X-ray dose by adjusting the voltage to the appropriate range. For instance, Matsumoto *et al*. found that the least noise in single-source CT scans occurred at 69 keV [11].

Slice thickness is another important factor influencing the quality of images. It is determined by the collimator settings, and its values are determined by the operator based on the clinical examination requirements, typically ranging from 0.625 to 10 mm [12]. CT scans commonly utilize slice thicknesses within this range to strike a balance between spatial resolution (better in thin slices) and scan time for a particular area (shorter in thicker slices) [13]. In thin slices, the partial volume artifact decreases while the patient dose, image noise, and scan time increase. Conversely, the thicker the slices, the more photons are available, improving the signal-to-noise ratio (SNR) [14, 15]. However, thicker slices may compromise spatial resolution along the Z-axis. Image noise shows a linear relationship with changes in slice thickness [16, 17]. Thinner slice thickness decreases image noise, increases visibility, and improves diagnosis. Therefore, the balance between the slice thickness with the diagnostic content and image noise must be considered [18].

Previous studies have shown that the slice thickness increases the detail and structure of the CT image [12, 19]. Therefore, as the thickness of the slices decreases, the image noise decreases [18, 19]. Tamura *et al*. [3] highlighted the use of NRS software for modulating image noise and improving CT scan image quality. Various studies have proposed different software solutions for image reconstruction and enhancement. These software programs process the captured projections and apply reconstruction and editing algorithms to generate the final image. Processing methods are applied in spatial and frequency domains, including special filtration algorithms that smooth the image and reduce noise [8, 18]. This study aimed to investigate the impact of slice thickness on reducing image noise and increasing image quality in single-source CT scans.

## MATERIAL AND METHODS

### Study design and setting

A comparative study was conducted at the Rosz Halat emergency department in Erbil, Kurdistan region of Iraq, from 19 Feb 2022 to 12 May 2022. The study included 15 patients (10 males and 5 females) with an age range of 7 to 95 years old. The objective was to evaluate the effect of slice thickness on image noise using the Linux operating system and statistical analysis, aiming to improve diagnosis according to the single-source computed tomography (SSCT) head instructions.

### Evaluation of images number

The number of images per slice thickness is one of the factors influencing the quality of CT images. Following the protocol instructions, the number of images is higher for thin slices and decreases as the slice thickness increases. Accordingly, this study investigated the effect of the number of images on the quality of CT images to determine how changes in the number of images would affect image quality.

### CT scan procedure

To assess the effect of slice thickness on image noise, CT scans were performed with varying thicknesses ranging from 0.625 to 10 mm, using the instructions (Pitch 0.969:1, speed = 19.13 mm/rot, mA = 259/DR40). Images were obtained by scanning the Catphan700 Phantom CTP712 module. The region of interest (ROI) of 1000 mm2 was selected, and pixel values were measured for all DICOM (Digital Imaging and Communications in Medicine). The CT images were analyzed to determine the optimal thickness for reducing image noise while maintaining image quality. The Linux operating system was used to analyze DICOM CT images.

### Statistical analysis

**Figure 1:**
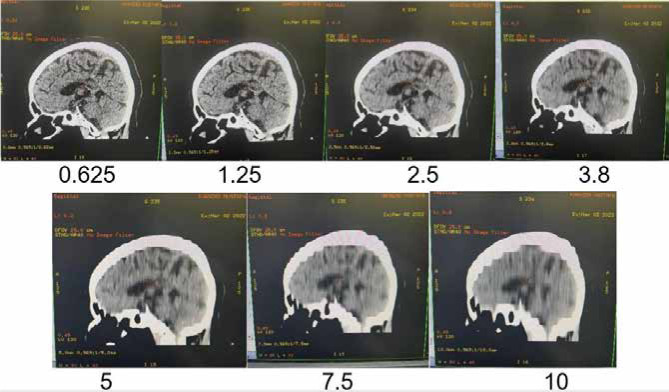
CT scan images with different thicknesses

F-test statistical analysis was performed to compare the significance of the variance of the obtained data. This test is used to determine the significance of variance between two groups of samples created depending on the change in slice thickness. The single-source CT scan of the head protocol was performed with slice values ranging from 0.625 to 10 mm. The CT scan was performed using a single-source CT scanner in spiral scanning mode with a voltage range of 9.1 to 6.7 kVp (Figure 1). Parameters related to kVp (X-ray strength or quality), mAs (number or amount of X-rays), and time (duration of radiation exposure) were also examined. The parameters used in the single-source CT scan of the head protocol are presented in Table 1.

**Figure 2:**
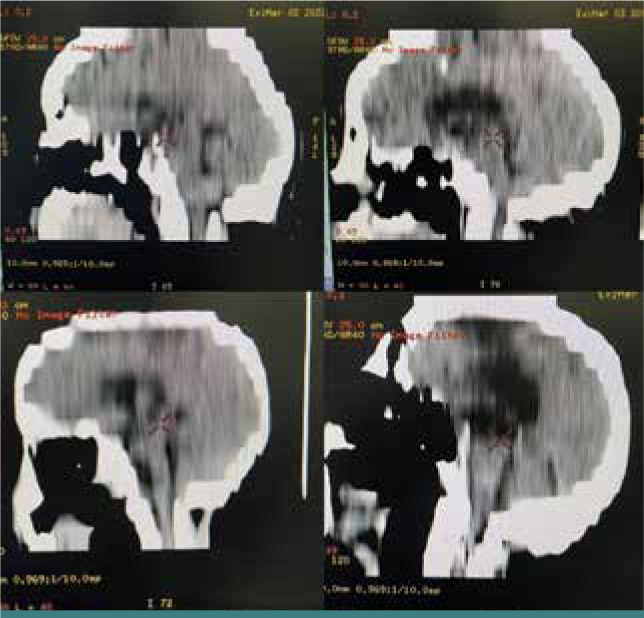
CT scan images with different pixel values

**Table 1:** Single-source CT scan of head parameters

Parameter	Protocol
Scan mode	Helical
mAs	1.9 to 7.6 kVp
Pitch	0.969:1
Slice thickness (mm)	0.625 to 10 mm
Exposure time	Range of (8.37 to 11.16)
Speed	19.13 mm/rot
No. of image	18 to 258

### Image review and analysis

Image review was performed using Image J software. The pixel intensities of the Catphan 700 phantom images were measured at different slice thicknesses. Statistical analysis was performed using the F-test function. The F-test function was used to compare statistical noise between images at different slice thicknesses. Further, the F-test was used to determine the significance of variance between the two groups of samples created depending on the change in the slice thickness since the variance and the standard deviation are the criteria that indicate the scatter of a distribution.

## RESULTS

The mean and standard deviation using different values of slice thickness are presented in Table 2.

**Table 2:** Main statistical measurements for different slice thickness values using single-source CT scan of head protocol

Technique	Slice thickness (mm)	Mas	Mean	Standard deviation	Variance
Head	0.625	1.6	89.251	6.525	32.876
Head	1.25	5.37	86.341	4.781	22.294
Head	2.5	3.8	85.251	3.821	13.443
Head	3.75	3.1	87.521	3.313	6.210
Head	5	2.69	85.015	2.295	3.782
Head	7.5	2.19	89.781	1.752	2.574
Head	10	1.9	87.421	1.549	2.481

**Figure 3:**
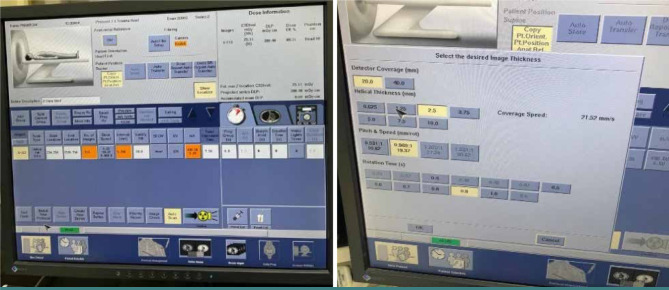
Reduction effect of slice thickness on noise

Based on the image analysis, there is a linear relationship between image noise and slice thickness. As the slice thickness decreases, the image noise also decreases. Moreover, the results show an inverse relationship between slice thickness and the number of photons (mAs). Increasing the slice thickness reduces the number of photons received, leading to a decrease in variance and standard deviation and an increase in the SNR of the image (Figure 3).

According to the present study, an increase in the slice thickness simultaneously affects the two factors of noise and diagnostic content of CT images. Accordingly, a decrease in the thickness reduces the noise and CT detection power. A reduction in the thickness causes more contrast, and the differentiation of small lesions is significantly improved due to the reduction in the partial volume effect. Therefore, the slice thickness reduction results in an increase in the diagnostic value of CT. For this reason, the lowest slice thickness with less image noise is considered the best value.

The test results indicate a significant difference between the slice thickness of 0.625, 1.25, and 2.5. However, no statistically significant difference was observed among slice thicknesses of 3.75, 5, 7, and 10. The values of F were less than the critical value of F (3.143) in the range of 3.75 to 10 mm, indicating no statistically significant difference in image noise among these slice thicknesses. The lower the slice thickness, the higher the diagnostic value. The slice thickness of 0.625 mm was identified as the best for reducing image noise and achieving better and more accurate detection using the single-source CT scan of the head protocol (Table 3).

**Table 3:** Results for slice thickness values (3.75, 5, and 7)

Slice thickness (mm)	Another Slice thickness (mm)	F-value	F-critical value	p-value
3.75	5	1.679	3.143	0.784
	7.5	1.743	3.143	0.754
	10	1.857	3.143	0.698
5	7.5	1.121	3.143	0.845
	10	1.197	3.143	0.921
7.5	10	1.375	3.143	0.723

Finally, as shown, the change in variance and standard deviation is most pronounced at 1.25 mm, which is commonly used for diagnostic purposes. Moreover, 0.625 mm provided high-resolution images with the most accurate diagnostic value, while 2.5 mm yielded an acceptable change compared to other thickness values. These results are consistent with F-test measurements, and it can be concluded that there was no significant image noise in the range of 1.25 to 2.5 mm.

**Figure 4:**
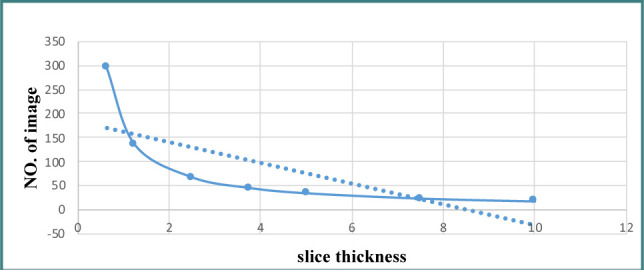
Relationship between slice thickness and number of images

The results showed that the number of images decreased with an increase in thickness. This increase in thickness was associated with a decrease in image quality, and finally, a thinner slice thickness was accompanied by an increase in quality. The diagram in Figure 3 shows this relationship, with the dotted line representing the relationship between image quality (Figure 4).

## DISCUSSION

The current study evaluated the effect of slice thickness on image noise using Image J software and statistical analysis to improve diagnosis according to the single-source CT scan of the head instructions.

The findings of this study are consistent with previous research that explored the relationship between noise and slice thickness, demonstrating that thinner slices result in better image quality and higher diagnostic value. Muhler *et al*. [15] investigated the effect of slice thickness on forensic diagnosis and identified a 1 mm slice as the most efficient for diagnosis. Jung *et al*. [20] conducted a quantitative analysis of three-dimensional imaging of the human skull using slice thicknesses of 1.25, 2.5, 3.75, and 5 mm to qualify CT performance. The results showed that thinner slices led to better image quality.

Image quality in computed tomography (CT) remains a major concern in clinical research and correct diagnosis. It is affected by physical factors of the scanner, such as the X-ray tube and other factors, including tube voltage (kVp), tube electrical current (mA), and slice thickness. All these parameters create some noise in the generated image [16]. Manson *et al*. [17] investigated the relationship between noise changes, as well as mAs and slice thickness. Their results showed an inversely proportional relationship between the noise ranges. In addition, they indicated that mAs and noise decreased with the decrease in slice thickness, in line with the results of the present study. Moreover, Alshipli *et al*. [21] used the DECT head instructions to determine the best thickness that reduces image noise. Their results indicated significant differences in image noise between thicknesses of 0.6, 1.25, and 2.5 mm compared to thicknesses of 3, 4, 5, and 6 mm. While their investigation identified 3 mm as the best slice thickness, this study concluded that 1.25 mm provided the optimal balance of reduced image noise and improved diagnostic accuracy in single-source CT scans. Limitations of this study include the small sample size. Therefore, it is recommended to replicate this investigation on a larger number of CT scans to further validate the findings.

## CONCLUSION

In this study, the changes in noise associated with slice thickness were investigated. The findings can serve as a reference for quality control methods in CT scan centers. The Linux operating system shows that image noise decreases with reduced slice thickness. Thus, a significant difference was observed in the noise of DICOM images obtained at thicknesses above 3.75 mm. Since the diagnostic value increases with decreasing slice thickness, the 1.25 mm slice thickness was considered the best value, reducing image noise with sufficient diagnostic value in the single-source multi-slice computerized axial tomography of the head protocol.

## Data Availability

Data supporting the findings of this study will be made available to other researchers upon reasonable request.

## Personal thanks

We would like to thank all the parents of the patients who generously contributed their time and support to this research. Their cooperation and patience were instrumental in carrying out this study. Additionally, we extend our gratitude to the Vice Chancellor for Research of Erbil University for their valuable assistance and support throughout the project, which greatly facilitated the completion of this project.
